# Potential Role of Dipeptidyl Peptidase IV in the Pathophysiology of Heart Failure

**DOI:** 10.3390/ijms16024226

**Published:** 2015-02-16

**Authors:** Thiago A. Salles, Leonardo dos Santos, Valério G. Barauna, Adriana C. C. Girardi

**Affiliations:** 1 Laboratory of Genetics and Molecular Cardiology, Heart Institute (InCor), University of São Paulo Medical School, São Paulo 05403-000, SP, Brazil; E-Mail: sallesth@gmail.com; 2 Department of Physiological Sciences, Federal University of Espírito Santo, Vitoria 29043-900, ES, Brazil; E-Mails: leodossantos@hotmail.com (L.S.); valerio.barauna@ufes.br (V.G.B.)

**Keywords:** glucagon-like peptide-1, brain natriuretic peptide, stromal cell-derived factor-1, renal function, cardiac dysfunction, natriuresis, cardioprotection, renoprotection

## Abstract

Dipeptidyl peptidase IV (DPPIV) is a widely expressed multifunctional serine peptidase that exists as a membrane-anchored cell surface protein or in a soluble form in the plasma and other body fluids. Numerous substrates are cleaved at the penultimate amino acid by DPPIV, including glucagon-like peptide-1 (GLP-1), brain natriuretic peptide (BNP) and stromal cell-derived factor-1 (SDF-α), all of which play important roles in the cardiovascular system. In this regard, recent reports have documented that circulating DPPIV activity correlates with poorer cardiovascular outcomes in human and experimental heart failure (HF). Moreover, emerging evidence indicates that DPPIV inhibitors exert cardioprotective and renoprotective actions in a variety of experimental models of cardiac dysfunction. On the other hand, conflicting results have been found when translating these promising findings from preclinical animal models to clinical therapy. In this review, we discuss how DPPIV might be involved in the cardio-renal axis in HF. In addition, the potential role for DPPIV inhibitors in ameliorating heart disease is revised, focusing on the effects of the main DPPIV substrates on cardiac remodeling and renal handling of salt and water.

## 1. Introduction

Heart failure (HF) is a complex syndrome characterized by the inability of the heart to pump sufficient amounts of blood to the circulation, or it can only do so by elevating ventricular filling pressures. The current pathophysiological concept of this syndrome is complex and involves a progressive disorder consisting of ventricular remodeling and inflammatory and neurohormonal responses, resulting from single or multiple causal events, which culminate in fatigue, dyspnea, exercise intolerance and fluid retention [1,2,3]. Although the etiologic keystones of HF can be diverse, diseases such as hypertension, myocardial infarction and diabetes are important risk factors. Taking into account that cardiac diseases are the leading cause of mortality in the modern world and that the prevalence of HF increases considerably with age, it is expected that HF will continue to be an important health and economic burden [4]. Such aspects justify the effort to obtain a better understanding of the HF syndrome, particularly with regard to enabling the development of novel therapeutic and preventive approaches.

Dipeptidyl peptidase IV (DPPIV), also known as CD26, is a widely expressed serine peptidase that exists on the surface of various cell types; however, its expression level differs greatly among cells. High levels of DPPIV-mRNA and abundant protein levels are found in the kidneys, small intestine and lung; moderate levels exist in the pancreas, liver and spleen; low levels are found in the stomach and heart, and no detectable expression exists in the brain and skeletal muscles [5]. The kidney is the main source of DPPIV, where it is one of the major brush border membrane proteins [6]. Within the kidneys, DPPIV is also present in the glomerular podocytes and capillaries [7]. In the systemic vasculature, DPPIV is expressed in the endothelial cells of venules and particularly in the capillaries. In fact, in different organs and tissues such as the lung, muscle and heart, almost all tissue DPPIV activity is due to its presence in the microvasculature [7,8]. DPPIV is also found in cells of the hematopoietic system, especially those involved in the immune response such as T, B and NK cells [7]. In the immune system, DPPIV is associated with T cell signal transduction as a co-stimulatory molecule. Surprisingly, the co-stimulatory activity of DPPIV requires its peptidase activity [9], and only soluble DPPIV with peptidase activity can enhance the proliferative response of peripheral blood lymphocytes [10,11]. Interestingly, DPPIV knockout mice display reduced plasma levels of interleukin-2, interleukin-4, IgG, IgG1, IgG2a and IgE after pokeweed mitogen (PWM) immunization [12].

Notably, a soluble form of DPPIV (sDPPIV) can be found in plasma and other body fluids [7,13]. There are very few studies available in the literature concerning the origin of sDPPIV. Some studies support the notion that sDPPIV is generated from cleavage of the DPPIV expressed at the membrane of peripheral lymphocytes, especially T lymphocytes, through the catalytic action of a yet unidentified “sheddase” (*i.e.*, an enzyme that cleaves the extracellular portion of transmembrane proteins, releasing them into the extracellular medium) [7,14]. Accordingly, some reports have shown that expression of DPPIV in lymphocytes is decreased in pathological states in which the activity and serum DPPIV abundance is high [15,16]. Circulating DPPIV activity is increased in obese patients, and indeed, adipose tissue has also been recognized as a source of sDPPIV [17,18]. In this regard, Röhrborn and colleagues [19] reported that the interplay among different metalloproteases is involved in constitutive DPPIV shedding from adipocytes and that the enzymes that mediate this posttranslational modification of DPPIV may act in a cell type-specific manner.

Transmembrane and soluble forms of DPPIV preferentially cleave dipeptides from the amino terminus of polypeptides with a proline or alanine at the second position [20]. DPPIV catalyzes the release of dipeptides from numerous substrates with known biological effects, including hormones, chemokines, neuropeptides and growth factors [7]. The most widely studied DPPIV substrate is incretin hormone glucagon like peptide-1 (GLP-1), which plays a pivotal role in the maintenance of systemic glucose homeostasis. In 2000, a seminal study by Marguet and colleagues [21] showed that the circulating intact insulinotropic form of GLP-1 [22] is preserved in DPPIV knockout mice and that specific genetic deletion or pharmacological inhibition of DPPIV improves insulin secretion and glucose tolerance. Not long after that, the first DPPIV inhibitor, sitagliptin, was approved by the FDA for managing glucose homeostasis in type II diabetic patients. Currently, seven DPPIV inhibitors, known as gliptins, have been approved for use as anti-diabetic drugs worldwide.

In addition to its exopeptidase activity, DPPIV also functions as a binding protein. In the renal proximal tubule, DPPIV interacts with the Na^+^/H^+^ exchanger isoform 3 protein (NHE3) [23]. NHE3 plays a critical role in sodium reabsorption, extracellular volume homeostasis and blood pressure control [24]. DPPIV inhibition reduces NHE3 activity *in vitro* and *in vivo* [25,26], underscoring the possible role of DPPIV in fluid retention. Moreover, DPPIV directly binds to collagen [27,28], and fibronectin [29,30]. In fact, together with seprase, DPPIV forms a protease complex that contributes to cell migration and repair of connective tissue [31]. Interestingly, DPPIV inhibition has been shown to attenuate cardiac fibrosis in HF rats [32,33,34] as well as in other models of cardiac disease [35,36,37,38]. It is therefore tempting to speculate that an association of DPPIV with collagen and/or fibronectin may be involved in cardiac tissue remodeling, but this assumption requires further investigation.

HF is characterized by cardiac dysfunction, increased renal vascular resistance and sodium retention. The findings that DPPIV catalytic activity, as well as its binding properties, are associated with increased sodium reabsorption [26,39,40], inflammation [41,42,43] and cardiac fibrosis [32,33,36,37,38] are consistent with the hypothesis that increased DPPIV activity plays a role in the pathophysiology of HF. In this review, we discuss how DPPIV might be involved in the cardio-renal axis of HF. Furthermore, the potential role for gliptins in ameliorating heart disease is revised, focusing on the effects of the main DPPIV substrates on cardiac remodeling and renal handling of salt and water.

## 2. Dipeptidyl Peptidase IV (DPPIV) and Cardiac Dysfunction

Emerging evidence from both preclinical and clinical studies raises the possibility that DPPIV might be involved in the pathophysiology of HF. After a six-month follow-up period, patients with episodes of acute HF that were discharged with the highest circulating DPPIV levels (highest quartile) displayed a BNP-independent three-fold higher risk of death due to HF within six months [44]. In line with these findings, we and others have found that HF patients [33] and animal models [33,34,45], exhibit increased DPPIV plasma activity compared to controls, and DPPIV activity is negatively correlated with the left ventricular ejection fraction and pulmonary congestion [33]. Of note, plasma DPPIV activity seems to be increased independently of the etiology of HF because patients with different causes of HF were included in the study [33]. Furthermore, in patients with diastolic dysfunction, the higher the activity of DPPIV in the coronary sinus and peripheral circulation, the poorer the diastolic function [34].

Interestingly, in addition to higher circulating enzymatic activity, HF rats may also exhibit elevated DPPIV activity and protein abundance in the heart. In a left ventricle radiofrequency ablation model of HF [33], cardiac activity and the expression of DPPIV, confined mainly to endothelial cells, were increased compared to sham-operated rats [33]. Additionally, Shigeta *et al.* [34] found that streptozotocin (STZ)-induced diabetic rats with cardiac dysfunction exhibit increased cardiac DPPIV activity and expression. Conversely, these same authors demonstrated that cardiac DPPIV activity and expression were reduced compared to controls in a model of pressure overload-induced HF [34]. Whether these conflicting results are due to the different models of myocardial injury-induced HF remains to be clarified. In fact, *in situ* regulation of DPPIV in HF seems to be a complex issue. Although the kidney is the organ with the highest expression level of DPPIV, HF animals do not show an increase in DPPIV in the kidneys, suggesting that this enzyme is transcriptionally and/or post-transcriptionally regulated in an organ specific manner. Notably, the downstream effectors protein kinase A (PKA) and protein kinase G (PKG), which are activated by the DPPIV substrates GLP-1 and BNP, respectively, were downregulated in the kidneys of HF rats [33]. These observations suggest that the soluble form rather than renal DPPIV is responsible for mitigating the natriuretic actions of GLP-1 and BNP in HF animals.

The molecular mechanisms and stimuli mediating the increase in the activity and abundance of both soluble and cardiac DPPIV in HF remain unresolved. An intriguing finding with regard to the modulation of DPPIV expression in HF is that competitive inhibition of DPPIV by sitagliptin also reduces DPPIV abundance both in the plasma and the heart [33]. A possible explanation for this unexpected observation arose from a study by Kanasaki and colleagues [46], which demonstrated that linagliptin increases the expression of components of the microRNA (miRNA) 29 family, which in turn reduce DPPIV abundance in the kidneys and endothelial cells of STZ-induced diabetic mice. Interestingly, the miRNA 29 family is downregulated after myocardial infarction [47,48]; however it remains to be established whether this posttranscriptional mechanism is also involved in the up-regulation of DPPIV activity/expression in the heart of experimental models of HF. Moreover, it is tempting to speculate that the sheddase that releases DPPIV from the cell surface to the bloodstream is stimulated in HF as well as other cardiovascular and metabolic diseases in which serum DPPIV activity is greater than that of healthy individuals.

Some evidence from *in vitro* studies indicates that DPPIV expression and activity can be activated by HF-related stimuli. HF can be considered as a chronic pro-inflammatory state [1] in which cardiac remodeling and dysfunction correlates with increased AGEs and AGE receptors [49,50]. In this context, various inflammatory cytokines can increase DPPIV expression in immune, epithelial or endothelial cells [51,52,53,54]. Additionally, AGEs may up-regulate cellular DPPIV expression [55,56] and subsequently increase its release from the cell surface of endothelial cells [55]. Furthermore, hypoxia, which often occurs in HF as a result of impaired tissue blood supply, is also capable of up-regulating DPPIV in different cell types [19,57,58].

**Figure 1:**
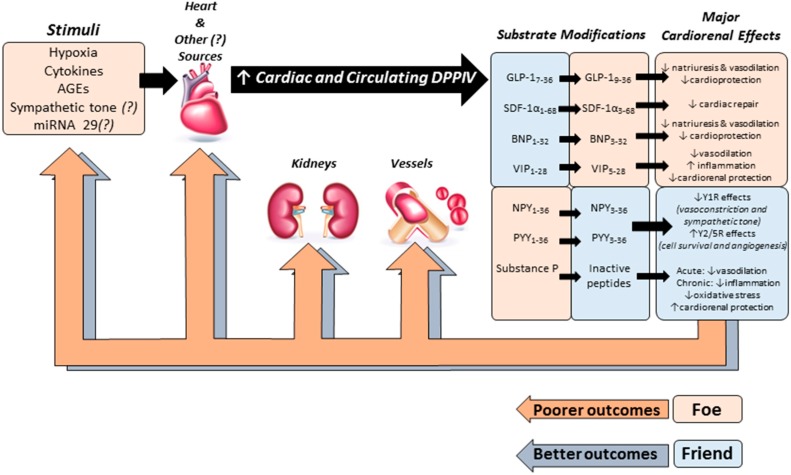
Schematic model depicting the possible role of DPPIV in the pathophysiology of HF. Several stimuli may increase the activity and abundance of both soluble and cardiac DPPIV in HF during the acute and/or chronic stages of this syndrome. High DPPIV activity may reduce the biological activity of peptides with cardio-, vaso- and renoprotective actions including glucagon-like peptide-1 (GLP-1), brain natriuretic peptide (BNP), and stromal cell-derived factor-1 α (SDF-1α) leading to poorer cardiovascular outcomes. On the other hand, the protease activity of DPPIV can be beneficial for the cardiovascular system by cleaving neuropeptide Y (NPY) and peptide YY (PYY).

Despite the uncertainties regarding how DPPIV activity and expression are upregulated in HF, elevated circulating levels of sDPPIV in HF have been consistently reported in pre-clinical and clinical studies [33,34,45]. High DPPIV activity most likely decreases the bioavailability of peptides with cardiorenal functions, and lower biological activity of these DPPIV substrates may be associated with progressive cardiac dysfunction and increased sodium and water retention by the kidneys (Figure 1).

## 3. Cardiorenal Effects of DPPIV Substrates

### 3.1. Glucagon-Like Peptide-1 (GLP-1)

GLP-1 is an incretin hormone secreted from intestinal L-cells in response to nutrient ingestion that potentiates glucose-dependent insulin secretion, suppresses glucagon levels and improves β-cell function [59]. Because native GLP-1 is rapidly degraded by DPPIV, its therapeutic use is limited. Thus, DPPIV inhibitors and GLP-1 receptor (GLP-1R) agonists that are resistant to DPPIV degradation have been developed and are currently in use as anti-diabetic agents [60,61].

In addition to its effect on glucose homeostasis, several independent lines of evidence have demonstrated that GLP-1 exerts beneficial renal and cardiovascular actions independent of its glucose-lowering actions [62,63,64,65,66]. The acute diuretic and natriuretic actions of GLP-1 have been consistently demonstrated by a variety of studies in rodents [67,68,69,70] and humans [71,72,73,74]. The mechanisms underlying the natriuretic effects of GLP-1 involve the inhibition of NHE3-mediated renal proximal tubule sodium reabsorption [68,69,70]. In fact, stationary *in situ* microperfusion experiments have demonstrated that GLP-1 is capable of directly inhibiting NHE3 via the cAMP/PKA signaling pathway [68]. GLP-1 may also be involved in increasing urinary sodium excretion through indirect mechanisms because the GLP-1R agonist liraglutide has been shown to induce atrial natriuretic peptide (ANP) secretion in mice [75]. Interestingly, in a double-blind, single-day study, GLP-1 infusion induced diuresis and natriuresis in healthy subjects; however, these renal effects were not accompanied by significant changes in plasma proANP concentrations [71]. The effects of GLP-1 on sodium and water homeostasis may also involve hemodynamic mechanisms because GLP-1 infusion is known to increase the glomerular filtration rate and renal plasma flow. DPPIV inhibitors also induce diuresis and natriuresis in rodents; however, the effects of DPPIV inhibition on renal sodium and water handling may occur through both GLP-1 dependent and independent mechanisms, given that infusion of a gliptin was capable of inducing natriuresis in GLP-1R knockout mice [69]. Notably, GLP-1 as well as GLP-1R agonists also confer renoprotection by reducing albuminuria and ameliorating renal damage in numerous experimental models of cardiovascular and renal diseases [76,77,78,79,80].

The cardioprotective actions of GLP-1 independent of glucose control have also been reported in both preclinical and clinical studies [65,81,82,83,84,85,86]. *In vitro*, GLP-1R agonists activate cytoprotective pathways and reduce cardiomyocyte apoptosis in response to diverse stimuli such as ceramide, palmitate, staurosporine and tumor necrosis factor-α [65,87]. Additionally, native GLP-1 attenuates infarct size after ischemia/reperfusion in *in vivo* and isolated perfused hearts [81], and liraglutide improves myocardial infarction (MI) outcomes in both diabetic and non-diabetic mice [83]. Furthermore, these preclinical results are supported by clinical data because GLP-1 and exenatide treatment significantly improves cardiac function and the myocardial salvage index in patients with acute MI and left ventricular dysfunction independently of the history of diabetes [82,85].

Interestingly, similar to DPPIV, GLP-1R is abundantly expressed in the vasculature, and GLP-1 has vasoactive properties. Vasodilatory actions of GLP-1 have been reported in several vessels as GLP-1 induces vasorelaxation in the aorta and the femoral, renal and pulmonary arteries [88,89,90]. More detailed information about the vascular properties of GLP-1 can be found in recent review articles [86,91].

### 3.2. Brain Natriuretic Peptide

BNP is produced in myocardial cells and secreted in response to distention of the cardiac chambers. Originally synthesized in the heart as the 108 amino acid precursor (pro-BNP)_1–108_, pro-BNP undergoes posterior processing, which culminates in the release of the biologically active form BNP_1–32_ and the *N*-terminal proBNP_1–76_ [92]. Active BNP_1–32_ binds to the natriuretic peptide-A receptor (NPR-A), which, via cyclic guanosine monophosphate (cGMP) and PKG, mediates its vasodilatory and natriuretic effects. Importantly, BNP is either cleared by the natriuretic peptide-C receptor (NPR-C) or degraded by neutral endopeptidase (NEP) or DPPIV [93].

BNP plays an important role in regulating extracellular fluid homeostasis and blood pressure by counteracting the actions of the sympathetic nervous system and the renin-angiotensin aldosterone system [94,95,96]. BNP exerts its natriuretic effects by both renal hemodynamic and tubular effects. In the glomerulus, BNP causes afferent arteriolar dilation together with efferent arteriolar vasoconstriction, thereby increasing the glomerular filtration rate (GFR). In the inner medullary collecting ducts, it decreases sodium chloride reabsorption, thereby increasing natriuresis [95]. Moreover, BNP also decreases aldosterone and renin release [94].

Plasma levels of BNP are increased in patients with HF and positively correlate with the degree of left ventricular dysfunction [97,98,99]. Indeed, BNP has been widely used as a reliable prognostic indicator for HF patients in all stages of the disease [100,101]. However, despite exceedingly high circulating levels of BNP measured by commercially available immunoassays, HF patients continue to experience fluid retention, increased peripheral vascular resistance and edema [102,103]. Several mechanisms have been proposed to explain the hyporesponsiveness to BNP in HF [103,104], including an increase in the proximal tubule sodium reabsorption with a resultant decrease of sodium delivery to the distal nephron where the BNP receptor is located, increased activity of peptidases that degrade and inactivate these peptides and/or decreased activity of peptidases that activate the peptides. Indeed, a report by Inoue *et al.* [105] demonstrated that NHE3 transport activity is significantly higher in the renal proximal tubules of an experimental model of post-myocardial injury-induced HF than in sham-operated animals. In addition, the endocrine BNP paradox has also been attributed to a deficiency of the active form of BNP in HF patients [106,107]. In fact, quantitative mass spectrometric analysis has demonstrated that the intact form of BNP is absent in the plasma of patients with severe chronic HF (New York Heart Association (NYHA) class IV) [106]. Interestingly, des-serine-proline BNP_3–32_, the cleaved form of BNP yielded by *N*-terminal dipeptide removal by DPPIV [108], displays remarkably reduced natriuretic actions and a lack of vasodilatory activity compared to BNP_1–32_ [109]. In line with these findings, overpacing-induced HF pigs treated with the DPPIV inhibitor sitagliptin exhibited an improvement in stroke volume and GFR. Moreover, acute BNP infusion was able to significantly improve end-systolic elastance, ventricular-arterial coupling and mechanical efficiency in HF pigs solely treated with sitagliptin [110].

### 3.3. Stromal Cell-Derived Factor 1-α (SDF-1α)

SDF-1α, also known as chemokine CXCL-12, is a potent chemoattractant protein that plays a fundamental role in leukocyte recruitment to inflammatory sites. SDF-1α effects are thought to be mediated mainly by binding to the G protein-coupled receptor CXCR4, although binding to CXCR7 has also been described [111]. Due to the prominent effects of this chemokine in leukocyte and stem cell recruitment to injury sites, several groups have studied its role after cardiac injury. It has been well described that after a cardiac injury, similar to MI, SDF-1α expression rapidly increases, and due to the higher gradient, several types of cells migrate to the injured heart tissue with the aim of improving cardiac repair and remodeling [111,112,113]. Among the cells that migrate to the injured heart tissue, bone marrow and circulating CXCR4^+^ progenitor cells are crucial for increasing cardiac angiogenesis and reducing cardiac remodeling [114]. Accordingly, several studies have shown that SDF-1α is a potent angiogenic factor *in vitro* [114]. Therapeutic use of SDF-1, in a similar manner to that of native GLP-1, is also challenged by its rapid degradation by DPPIV and matrix metalloproteinase-2. Indeed, a protease-resistant variant of SDF-1α significantly improves blood flow in a model of peripheral artery disease and exhibits greater potency for cardioprotection than wild-type SDF-1α after MI [113,115,116]. Moreover, synergism between granulocyte-colony stimulating factor and DPPIV inhibition significantly improves stem cell mobilization, angiogenesis, cardiac function and survival after MI in rodents [117]. Notably, co-treatment with the CXCR4 antagonist AMD3100 reverses the recruitment of CD34^+^/CXCR4^+^ cells into the heart and mitigates the improvement in cardiac function [118].

Elevated levels of total SDF-1α and low migratory activity of circulating progenitor cells were both independent predictors of death or repeat acute MI and new-onset HF in patients with acute MI [119,120]. Interestingly, four-week treatment with sitagliptin significantly increased the levels of circulating endothelial progenitor cells in type 2 diabetic patients [121]. Moreover, after adjusting for traditional cardiovascular risk factors, SDF-1α was associated with HF and all-cause mortality risk in Framingham Heart Study participants [120].

### 3.4. Other DPPIV Substrates and Potential Effects on the Cardiovascular System

Vasoactive intestinal peptide (VIP) belongs to the pituitary adenylyl cyclase activation polypeptide (PACAP)/glucagon superfamily that includes other DPPIV substrates such as GLP-1, glucose-dependent insulinotropic peptide (GIP) and PACAP. VIP is found in the gastrointestinal tract, peripheral and central nervous system, heart, lungs and kidney, as well as in the plasma. The beneficial effects of intact VIP consist of vasodilatation-mediated increases in local blood flow, anti-inflammatory and anti-oxidative actions in ischemic organs [58], positive inotropic and chronotropic effects associated with coronary vasodilatation [122,123], and renoprotective effects by the suppression of oxidative stress [124] as well as diuresis and natriuresis [125]. VIP is cleaved by DPPIV in two consecutive steps, thereby producing truncated forms with reduced biological actions [126]. Thus, DPPIV inhibition and the consequent increased bioavailability of endogenous VIP may have beneficial effects in cardiovascular diseases including HF.

In addition to plasma metabolization by angiotensin converting enzyme (ACE), substance P is also inactivated by DPPIV. This neuropeptide, found primarily in sensory nerves, is also present in heart nerve fibers surrounding cardiac muscle cells, endocardium, epicardium and coronary vessels, as well as in coronary endothelial cells themselves [127]. Regarding cardiovascular actions, substance P plays an important role in adverse myocardial remodeling during its long-term activation in non-ischemic HF, inducing cardiac inflammation, oxidative stress, apoptosis and changes to the extracellular matrix [127]. However, cumulative evidence suggests that at the acute phase of ischemia-reperfusion settings, substance P may confer cardioprotection mainly by increasing myocardial reperfusion due to NO release and coronary dilatation [128,129,130]. Thus, it remains unresolved whether substance P inactivation by DPPIV would render deleterious or cardioprotective effects in HF [127].

DPPIV also mediates the cleavage of the 36 amino acids neuropeptide Y (NPY) and peptide YY (PYY) to *N*-terminally truncated NPY_3–36_ and PYY_3–36_ forms, respectively [131]. Both of these peptides bind to at least six different G-protein coupled receptors, Y1–Y6 [132]. NPY is an abundant neuropeptide in the central and peripheral nervous system and plays an essential role in sympathetic tone and behavioral function. After binding to the Y1 receptor, NPY induces a potent vasoconstrictor effect, whereas binding to Y2 is implicated in inhibition of neurotransmitter release. In addition, binding to Y5 mediates the regulation of food intake. Moreover, Y2/Y5 receptor stimulation is involved in proliferation of smooth muscle and endothelial cells, angiogenesis and nitric oxide (NO) production [133,134]. Interestingly, after DPPIV cleavage, the truncated form, NPY_3–36_, displays higher affinity to the Y2 and Y5 receptors than to the vasoconstrictor receptor Y1 [132]. PYY is a gastrointestinal hormone secreted mainly by L-cells and plays a role in regulation of food intake. Similar to NPY, which is a product of DPPIV cleavage, PYY_3–36_, also has a higher affinity to the Y2 and Y5 receptors. Thus, as opposed to most of the peptides discussed above, it is likely that in the case of NPY and PYY, the protease activity of DPPIV would actually be beneficial for the cardiovascular system. Notably, NPY levels have been found to be elevated in HF patients and to correlate with tachycardia and left-sided HF [135,136].

## 4. DPPIV Inhibitors and HF: Preclinical Studies

Diabetic patients have a three-fold higher risk of developing HF compared to non-diabetic subjects [137]. Because DPPIV inhibition is an effective therapy for reducing blood glucose, it is reasonable to assume that if DPPIV inhibition improved cardiac function at all, this improvement would likely be secondary to blood glucose control. However, as mentioned above, increased levels of plasma DPPIV have been associated with poorer outcomes in HF animals and patients [33,34,45]. In addition, most peptides inactivated by DPPIV display beneficial cardiorenal functions, suggesting that inhibition of DPPIV may attenuate the development and/or progression of HF by mechanisms independent of glucose reduction. Accordingly, a large body of experimental data [32,33,34,38,87,138] has demonstrated that genetic deletion or pharmacological inhibition of DPPIV improves cardiovascular outcomes.

Sauvé *et al.* [138] have demonstrated that normoglycemic DPPIV knockout mice display increased survival after experimental myocardial infarction (MI). Because DPPIV knockout mice are resistant to the development of diabetes induced by streptozotocin (STZ) [21], these authors examined the cardioprotective effects of pharmacological DPPIV inhibition in STZ-diabetic wild type mice subjected to MI. Likewise, treatment with sitagliptin improved survival post-MI in diabetic mice, and acute DPPIV inhibition was also capable of improving recovery from heart ischemia/reperfusion injury in normoglycemic mice. Additionally, four-week treatment with vildagliptin improved cardiac dysfunction, decreased fibrosis, attenuated cardiac levels of apoptosis and increased the survival rate in pressure-overloaded nondiabetic mice and rats [32,38]. Similarly, six-week treatment with sitagliptin significantly attenuated cardiac dysfunction in normoglycemic rats subjected to myocardial injury by radiofrequency ablation [33]. The preventive effects of inhibition of DPPIV included a reduction in diastolic left ventricular end pressure, increased systolic performance and decreased stiffness of the heart chamber. Treatment with sitagliptin also attenuated cardiac hypertrophy and interstitial fibrosis of the remaining myocardium. Furthermore, inhibition of DPPIV was able to prevent a decrease in the glomerular filtration rate and an increase in NHE3-mediated proximal tubular reabsorption of sodium and minimized pulmonary congestion [33]. On the other hand, Yin and colleagues have found that administration of the DPPIV inhibitor vildagliptin (15 mg/kg/day) failed to prevent cardiac remodeling and dysfunction after MI in rats [139]. Notably, the lack of vildagliptin-induced cardioprotection in this study is most likely attributed to the low dose and frequency of administration of vildagliptin employed (once-daily *vs.* twice-daily dosing frequency). Indeed, we have found that chronic treatment with high (120 mg/day given twice per day) but not low-dose (20 mg/kg given once a day) vildagliptin is capable of ameliorating cardiac and renal function and reducing pulmonary congestion in rats with established HF [140].

Taken together, these data suggest that DPPIV inhibitors might have a place in the therapeutic armamentarium for cardiovascular diseases other than diabetes.

## 5. DPPIV Inhibitors and HF: Clinical Studies

Despite the extensive amount of experimental data documenting that DPPIV inhibitors are beneficial for treating cardiac disorders, conflicting results have been found when translating these promising findings from preclinical animal models to clinical therapy.

In accordance with the pre-clinical studies, small pilot studies have reported positive effects of DPPIV inhibitors in patients with cardiac disease. In a small study, fourteen patients with coronary artery disease and preserved left ventricular function awaiting revascularization received an oral load of 75 g of glucose after a single dose of 100 mg of sitagliptin or placebo. Dobutamine stress echocardiography was conducted with tissue Doppler imaging at rest, during peak stress, and after 30 min of recovery. Interestingly, patients treated with the DPPIV inhibitor exhibited an improvement in global left ventricular function at peak stress, and after a 30 min recovery. Moreover, sitagliptin mitigated post-ischemic stunning dramatically compared to the placebo [141]. Because an oral load of glucose was administered to patients in this study, one can infer that GLP-1 may be the major DPPIV substrate responsible for the observed cardioprotective effects.

The failing heart undergoes an intense metabolic remodeling, switching its primary energy substrate to glucose. In this regard, DPPIV inhibition seems to exert a positive effect on myocardial energy metabolism because four-week treatment with sitagliptin significantly increased myocardial glucose uptake in a cohort of nondiabetic patients with nonischemic dilated cardiomyopathy [142]. These findings may be attributed, at least in part, to the fact that sitagliptin is capable of increasing the protein and mRNA expression of glucose transporter-4 (GLUT-4) in the heart [143], at least in part, due to a GLP-1-dependent mechanism because this incretin directly enhances GLUT4 expression in isolated cardiomyocytes *in vitro* [143].

Since 2008, regulatory agencies have demanded that all new anti-diabetic drugs undergo cardiovascular safety assessments. In 2013, two major clinical trials assessing the benefits and risks of DPPIV inhibitors in high-cardiovascular risk patients with diabetes had their results published in The New England Journal of Medicine: The Saxagliptin Assessment of Vascular Outcomes Recorded in Patients with Diabetes Mellitus—Thrombolysis in Myocardial Infarction 53 study (SAVOR-TIMI 53) [144] and the Examination of Cardiovascular Outcomes with Alogliptin *vs.* Standard of Care (EXAMINE) [145].

The SAVOR-TIMI 53 study was a multicenter, randomized, double-blind, placebo-controlled, phase 4 trial. A total of 16,492 patients with a history of documented type 2 diabetes mellitus, a glycated hemoglobin level of 6.5% to 12.0%, and either a history of established cardiovascular disease or multiple risk factors for vascular disease were randomly assigned to receive the DPPIV inhibitor saxagliptin at a dose of 5 mg daily (or 2.5 mg daily in patients with an estimated GFR of ≤ 50 mL/min) or a placebo. The primary endpoints consisted of cardiovascular death, nonfatal MI or nonfatal ischemic stroke. The secondary endpoints included hospitalization for HF, coronary revascularization, or unstable angina. The median follow-up period was 2.1 years. As expected, patients treated with saxagliptin exhibited lower levels of fasting plasma glucose and glycated hemoglobin. Notably, the saxagliptin group presented a better albumin-to-creatinine ratio than the placebo group, suggesting a positive effect on renal function. Unexpectedly, this trial showed a 27% increased relative risk of hospitalization for HF in patients assigned to the saxagliptin group (3.5% *vs.* 2.8% in placebo; *p* = 0.007) [144]. Further analysis showed that patients with a high overall risk of HF (*i.e.*, a history of HF, impaired renal function, or elevated baseline levels of *N*-Terminal proBNP) were more susceptible to the detrimental effects of the DPPIV inhibitor [146].

The EXAMINE trial was a multicenter, randomized, double-blind trial [145]. Unlike the SAVOR-TIMI 53 study, patients were eligible for enrollment if they had type 2 diabetes mellitus, a glycated hemoglobin level of 6.5% to 11.0%, and had an acute coronary syndrome within 15 to 90 days before randomization. Acute coronary syndromes included acute MI and unstable angina requiring hospitalization. The patients were assigned to receive alogliptin or a placebo. Because alogliptin is cleared by the kidneys, dose adjustment in patients with diabetes and chronic kidney disease was required. Patients with normal renal function or mild renal insufficiency, *i.e.*, levels of estimated GFR (eGFR) > 60 mL/min received 25 mg, patients with an eGFR of 30 to less than 60 mL/min received 12.5 mg and patients with an estimated GFR < 30 mL/min received 6.25 mg. The mean follow-up was 18 months, and the primary outcomes were cardiovascular death, nonfatal MI and nonfatal stroke. A total of 5380 patients were evaluated, and similar to the SAVOR-TIMI 53 study, no significant differences in primary cardiovascular outcomes between the placebo and alogliptin groups were observed [145]. Further analyses regarding HF and the EXAMINE trial were published, and despite the similar history of HF in both groups, alogliptin neither induced new onset of HF nor worsened the outcomes in patients with prior HF [147].

An ongoing study evaluating DPPIV inhibitors and cardiac outcomes is the Vildagliptin in Ventricular Dysfunction Diabetes (VIVIDD) trial. In the VIVIDD trial, 254 patients with type 2 diabetes mellitus, a glycated hemoglobin of 6.5% to 10%, and chronic HF (New York Heart Association class I to III) were randomized to receive vildagliptin (50 mg bid) or a placebo [148]. The ejection fraction (primary endpoint) was measured at baseline and after 52 weeks of follow-up. No significant difference in the ejection fraction was found between the groups; however, patients taking vildagliptin exhibited a significant increase in left ventricular end-diastolic volume, end systolic volume and stroke volume. Interestingly, despite the increased volume, after 52 weeks, BNP levels decreased by 14% relative to baseline in the placebo group *vs.* 28% in the vildagliptin group. These data suggest a decrease in cardiac stress. According to new findings reported at the American Diabetes Association 2014 Scientific Sessions, patients treated with the DPPIV inhibitor vildagliptin exhibit no significant difference in the incidence of hospitalization for HF compared to the placebo group [149].

The outcomes of two large ongoing studies are of high importance for clinicians and patients because they will help to clarify whether risks and/or benefits exist for DPPIV inhibitors used to treat type 2 diabetes patients with a history of cardiovascular disease. The Cardiovascular Outcome Study of Linagliptin *vs.* Glimepiride in Patients with Type 2 Diabetes (CAROLINA) study is a multicenter, randomized, parallel group, double blind study to evaluate the cardiovascular safety of linagliptin *vs.* sulfonylurea (glimepiride) in patients with type 2 diabetes mellitus at a high cardiovascular risk [150]. This trial has been ongoing since November 2010, and the estimated primary completion date is September 2018. Because it is such a large trial, it will provide a unique perspective with respect to cardiovascular outcomes and linagliptin.

Another important trial is the Sitagliptin Cardiovascular Outcome Study (TECOS) [151]. The TECOS is a multinational, randomized, double-blind, placebo-controlled trial designed to assess the cardiovascular outcome of long term treatment with sitagliptin in patients with type 2 diabetes mellitus, a history of cardiovascular disease and a glycated hemoglobin of 6.5% to 8.0%. It has been ongoing since November 2008 with an estimated enrollment of 14,000 patients and a primary completion date of December 2014.

## 6. Summary and Perspectives

**Figure 2:**
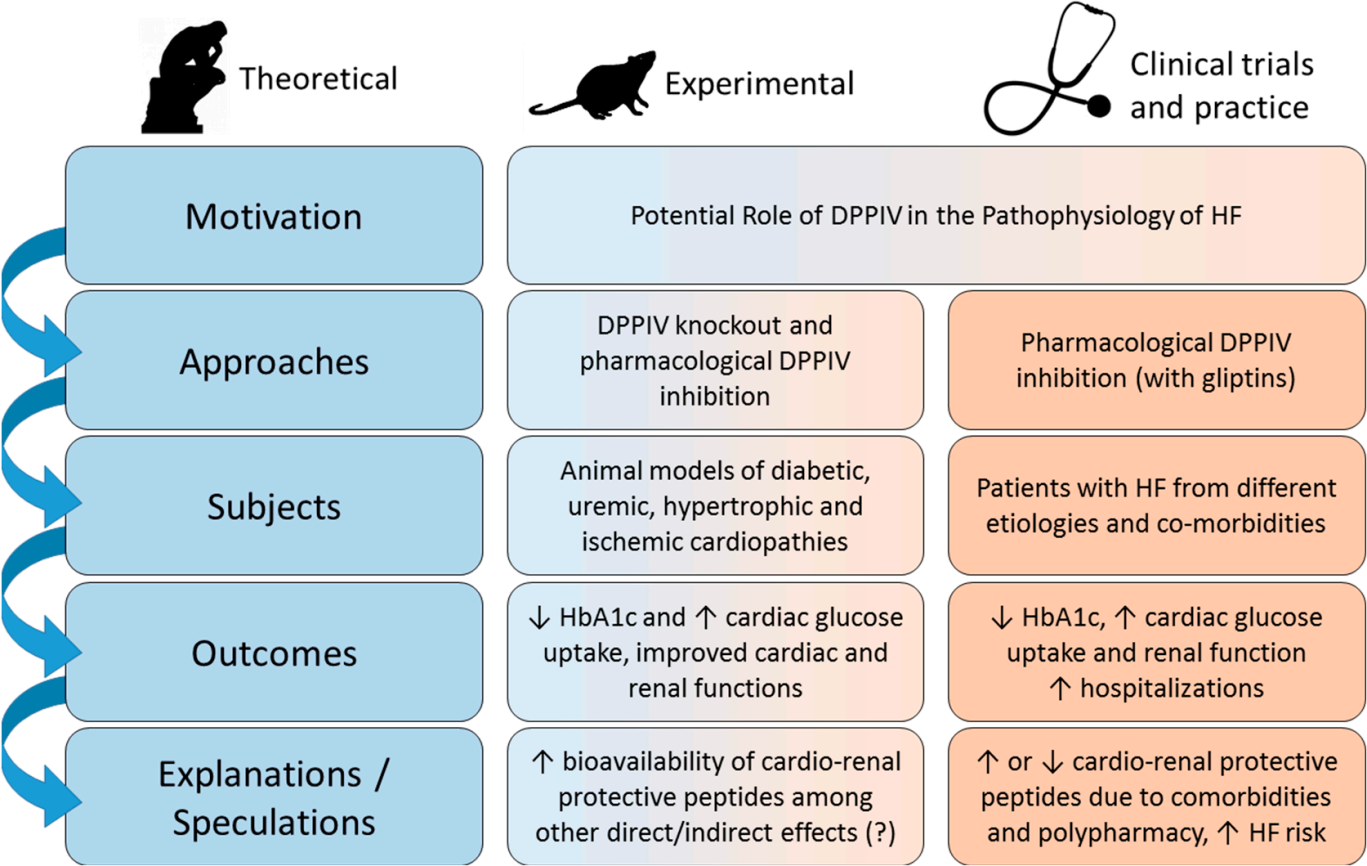
DPPIV as a therapeutic target in HF. Diagram summarizing the rationale and approaches used to test the hypothesis that DPPIV inhibition may exert cardio and renoprotective effects in experimental and clinical HF as well as the main outcomes obtained in experimental and clinical studies (see text for further detail).

The key points discussed in this review are summarized in Figure 2. Clinical and experimental studies have shown that the higher the activity of plasma DPPIV, the poorer the cardiovascular outcomes in HF, suggesting that DPPIV might be involved in the pathophysiology of this syndrome. The poor prognosis in the presence of high circulating levels of DPPIV is most likely due to the lower bioavailability of cardio and renoprotective peptides such as GLP-1, BNP and SDF-1α and to the fact that DPPIV directly and/or indirectly may exert pro-fibrotic and inflammatory actions. Thus, in theory, inhibition of DPPIV would confer cardioprotection. Indeed, the majority of preclinical studies have demonstrated that gliptins ameliorate cardiac remodeling and function and may even increase survival in experimental HF. It is of particular note that gliptins have been consistently reported to exert renoprotective actions in both experimental models of cardiac disease and in patients with cardiac dysfunction.

A lower level of consensus is evident with regard to the cardiovascular benefits of DPPIV inhibition in the clinical setting. Clinical studies have documented that DPPIV inhibitors may improve cardiac dysfunction in some patients, but not affect cardiac outcomes or even increase the risk of HF complications in others. In pilot studies, non-diabetic subjects with cardiac dysfunction treated with DPPIV inhibitors displayed improved myocardial glucose uptake, reduced circulating AGEs and elevated levels of circulating progenitor cells as compared to placebo-treated individuals. On the other hand, the results of the SAVOR-TIMI 53 and EXAMINE trials have shown that either saxagliptin or alogliptin, respectively, achieved non-inferiority, but not superiority, compared to placebo in terms of primary cardiovascular outcomes in type 2 diabetic patients. Actually, the increase in hospitalization for HF in diabetic patients treated with saxagliptin *vs.* patients treated with placebo observed in the SAVOR-TIME 53 trial have raised some cardiovascular safety concerns with respect to administering DPPIV inhibitors to diabetic patients with HF. It remains to be established whether this disappointing outcome can be considered an adverse effect of DPPIV inhibitors, what is its clinical relevance, whether it is common to all DPPIV inhibitors and if it may be associated with comorbidities and polypharmacy. So far, the possible underlying mechanisms to explain why saxagliptin would increase the risk for HF hospitalizations remain speculative.

Ongoing trials such as TECOS and CAROLINA may shed light on the potential benefits and drawbacks of DPPIV inhibition in diabetic patients with a history of cardiovascular diseases. Morever, together with additional translational research, the results from these clinical studies may also clarify whether DPPIV plays a role on the pathophysiology of HF beyond glycemic control in a harmful, beneficial or neutral way.
